# Dementia Adjudication Triggers Associated With Increased Mortality for Older Australians: Evidence From ASPREE

**DOI:** 10.1093/geroni/igaa057.523

**Published:** 2020-12-16

**Authors:** Jane Banaszak-Holl, Xiaoping Lin, Jing Xie, Stephanie Ward, Henry Brodaty, Raj Shah, Anne Murray, John McNeil

**Affiliations:** 1 Monash University, Melbourne, Victoria, Australia; 2 Peter Mac Cancer Centre, Melbourne, Victoria, Australia; 3 UNSW - Sydney, Sydney, New South Wales, Australia; 4 UNSW Sydney, Sydney, New South Wales, Australia; 5 Rush University, Chicago, Illinois, United States; 6 University of Minnesota, Minneapolis, Minnesota, United States

## Abstract

Research Aims: This study seeks to understand whether those with dementia experience higher risk of death, using data from the ASPREE (ASPirin in Reducing Events in the Elderly) clinical trial study. Methods: ASPREE was a primary intervention trial of low-dose aspirin among healthy older people. The Australian cohort included 16,703 dementia-free participants aged 70 years and over at enrolment. Participants were triggered for dementia adjudication if cognitive test results were poorer than expected, self-reporting dementia diagnosis or memory problems, or dementia medications were detected. Incidental dementia was adjudicated by an international adjudication committee using the Diagnostic and Statistical Manual for Mental Disorders (DSM-IV) criteria and results of a neuropsychological battery and functional measures with medical record substantiation. Statistical analyses used a cox proportional hazards model. Results: As previously reported, 1052 participants (5.5%) died during a median of 4.7 years of follow-up and 964 participants had a dementia trigger, of whom, 575 (60%) were adjucated as having dementia. Preliminary analyses has shown that the mortality rate was higher among participants with a dementia trigger, regardless of dementia adjudication outcome, than those without (15% vs 5%, Χ2 = 205, p <.001). Conclusion: This study will provide important analyses of differences in the hazard ratio for mortality and causes of death among people with and without cognitive impairment and has important implications on service planning.

